# Serological and histolomorphological investigation of camel bulls testes (*Camelus dromedaries*) during the rutting and non-rutting seasons

**DOI:** 10.1186/s12917-024-04108-6

**Published:** 2024-06-20

**Authors:** Fatma Ali, Ragab Hassan Mohamed, Mahmoud Abd-Elkareem, Mervat S. Hassan

**Affiliations:** 1 Physiology Department, Faculty of Veterinary Medicine, Aswan University, Aswan, Egypt; 2 Theriogenology Department, Faculty of Veterinary Medicine, Aswan University, Aswan, Egypt; 3 Department of Cell and Tissues, Faculty of Veterinary Medicine, Assiut University, Assiut, 71526 Egypt; 4 Theriogenology Department, Faculty of Veterinary Medicine, New Valley University, 725211 New Valley, Egypt

**Keywords:** Non-rutting, Rutting, Dromedary Camel, Oxidative stress, Antioxidant, Testes, Apoptosis, Spermatogenesis

## Abstract

**Background:**

Camels are bred for their milk, meat, wool and hair, transportation, and their excrement as fuel. The seasonal reproduction of camel bull is accompanied by changes in sexual activity, the morphology, and function of the testes. This study aimed to evaluate the seasonal fluctuations in serum testosterone (T) levels as well as total antioxidant capacity (TAC) and malondialdehyde (MDA) levels in the testes of dromedary bulls (*Camelus dromedarius*) during the rutting and non-rutting seasons. Moreover, the impact of rutting season on the testicular size and histomorphology was also observed. Seventy mature dromedary bulls were divided into a rutting group (*n* = 35) and a non-rutting group (*n* = 35). From these bulls, blood samples and testes were collected during the rutting season (October to April) and non-rutting season (May to September) from a local slaughterhouse.

**Results:**

All parameters changed significantly during rutting and non-rutting periods in camel bulls. The levels of TAC in testes, and serum T were significantly (*P* < 0.05) higher in the rutting group than in the non-rutting group. However, testicular MDA was significantly (*P* < 0.05) lower in the rutting group than in the non-rutting group. TAC was negatively correlated with MDA (*r* = -0.59, *p* < 0.01). Moreover, in the rutting group and the non-rutting group, T was positively correlated with levels of TAC (*r* = 0.66, *p* < 0.0003). Additionally, testicular size (length, breadth, and thickness) was significantly greater in camels during the rutting season than in camels during the non-rutting season. Moreover, the number and diameter of seminiferous tubules, and spermatogenesis increased during the rutting season, whereas, the collagen content and apoptosis increased during the non-rutting season.

**Conclusion:**

This study revealed that the rutting normal breeding season (NBS, rutting group) was associated with higher levels of total antioxidant capacity (TAC), T, and spermatogenic activity while the collagen content, concentrations of MDA (the oxidative stress factor) and apoptosis (an outcome of oxidative stress) were lower than those in the low breeding season (LBS, non-rutting group). In addition, the testicular size and seminiferous tubule diameter and number were higher during the NBS.

## Introduction

The onset and length of the normal breeding season (NBS; rutting season) in camels are related to breeding. In the Middle East, the sexually active period begins in late October and ends in late April [1, 2]. The seasonal reproduction of camel bull is accompanied by changes in sexual activity, the morphology, and function of the genital organs, as well as endocrine activation [1, 3]. As a seasonal breeder, the dromedary bull displays increased aggressiveness, protrusion of the soft palate, and profuse stinky fluid secretion from the poll glands [1, 4, 5]. During the rutting season, mature male dromedary camels exhibit increased testicular size and improved accessory gland activity [1, 3, 6]. In Egypt, the rutting season positively influences the weight of the ampullae, prostate, bulbourethral, and poll glands. Indeed, the concentrations of fructose and citric acid in the accessory glands of camels appear to be subject to seasonal variation, with the highest concentration occurring during the rutting season [1]. The process of spermatogenesis occurs throughout the year, and seasonality improves sexual behavior, libido and quantitative and qualitative sperm production [3, 7]. The lowest level of spermatogenesis coincides with the period of low breeding seasons (LBS; non-rutting season) [1]. During the non-rutting season, the number, size, and activity of Leydig cells decrease [8]. During the rutting season, testosterone levels and spermatogenesis are higher in male camels [7, 8]. In addition, testis weight and size significantly increase due to the development of interstitial tissues, and the concentration of testosterone is positively correlated with the duration of mating and ejaculated semen volume [6, 9].

The objective of the present study was to study variations in testosterone, testicular TAC, and MDA concentrations during the rutting and non-rutting periods and to provide additional information about the morphology and apoptosis of testicular cells during the rutting and non-rutting seasons.

## Materials and methods

### Animals

Seventy healthy camel bulls (*Camelus dromedarius*), aged 5 to 10 years, and with clinically normal reproductive organs were selected for this study. The animals used in this study were privately owned by other individuals and we obtained informed consent from the owners to use these animals in our study. The animals were slaughtered at the local abattoir in Aswan, Egypt and samples were collected during the rutting (winter and spring, *n* = 35) and the non-rutting seasons (summer and autumn, *n* = 35). The slaughter was carried out by an experienced technician by cutting the jugular vein with a sharp knife without using electrical stimulation or anesthesia. The animals were guaranteed to die before they could be processed any further or sampled. Testes were obtained from slaughtered camel bulls after they had been bled and skinned.

### Hormonal assay

Before slaughter, blood samples were collected from the jugular vein in plain vacuum tubes from 70 camel bulls. These samples were centrifuged at 1500 rpm for 10 min, after which the serum was collected and stored at − 20 °C. The concentration of testosterone in the serum samples were analyzed by ELISA using commercial kit (Bio Tina GmbH, 58,119 Hagen, Bugweg 53, Germany).

### Antioxidants assay

After slaughter, testes from camel bulls during the rutting (*n* = 35) and the non-rutting seasons (*n* = 35) were collected under hygienic conditions in a plastic bag containing normal saline, and transported to the laboratory (in an ice tank at 5 °C). Then the testes were washed with warm saline [10].

From each camel bull, one testis was individually squeezed using a metal squeezer for the collection of testicular fluid. The collected fluids were centrifuged (1500 x g for 10 min) and the supernatant was harvested and stored at 20 °C for analysis [11]. The concentrations of TAC and MDA levels were analyzed by commercial kits (Biodiagnostic, Cairo, Egypt) using a spectrophotometer (UV/VIS spectrometer -T80).

### Testicular measurement

Testicular measurements were carried out as previously described by [12]. Each of the testes was measured separately using a digital caliper to determine the height, length, and width of the testis [12].

### Histological examination

Testes were collected from apparently healthy slaughtered camels and immediately fixed in 10% neutral buffered formalin. The formalin-fixed testes were dehydrated in ascending grades of ethanol, cleared in methyl benzoate, and embedded in Paraplast. Paraffin Sect. 5 μm in thickness were cut and stained with the following histological stains:

1. Haematoxylin and Eosin for general histological examination of the testes [13].
2. Periodic acid Schiff (PAS) technique for demonstration of glycoprotein [13].
3. Crossmon’s trichrome technique for staining collagen fibers and connective tissue [14].
4. Picro-Sirius red technique to differentiate between mature and immature collagen fibers [15, 16].

All staining slides were examined by an Olympus BX51 microscope and the photographs were taken by an Olympus DP72 camera adapted into the microscope.

### TUNEL assay

The location of apoptosis was done utilizing *the In Situ* Cell Death Detection Kit, Fluorescein (Sigma-Aldrich). Terminal deoxynucleotidyl transferase (TdT) dUTP Nick-End Labeling (TUNEL) test was done to distinguish apoptotic cells that undergo extensive DNA fragmentation during the late stages of apoptosis. This method depends on the ability of TdT to label blunt ends of double-stranded DNA breaks independent of a template. The Protocol followed as per kit instructions and as the previously published protocol [17].

### Morphometrical measurements

Morphometrical measurements were done to know the diameter and numbers (per unit area) of seminiferous tubules in cross sections (profiles).

### Statistical analysis

The data were expressed as mean ± S.E. The data were subjected to statistical analysis using paired t-test Prism 5 (GraphPad Software). The significance value was set at (*P* < 0.05).

The relationships between testosterone, oxidant (MDA), and antioxidant (TAC) were statistically analyzed by Pearson correlation test. Pearson correlation coefficient (r) > 0.8 was considered highly correlated.

## Results

### Biochemical and biometrical analysis

The levels of TAC and MDA in testes of rutting and non-rutting camel bulls are shown in Table 1. The concentration of TAC in the testes of rutting bulls was greater (*P* < 0.05) than that of non-rutting bulls. However, MDA concentration in the testes was found to be lower (*P* < 0.05) in rutting bulls compared with non-rutting bulls. The levels of testosterone in the serum of rutting and non-rutting camel bulls are shown in Table 1. The serum testosterone concentration was higher (*P* < 0.01) during the rutting than during the non-rutting periods.

**Table 1:** TAC, MDA, and testosterone status of camel bulls in testis during rutting and non-rutting seasons

Parameters	Non-rutting	Rutting
**TAC (mmol/ml)**	0.60 ^b^ ± 0.05	1.80 ^a^ ± 0.05
**MDA (mmol/ml)**	2.10 ^a^ ± 0.03	0.98 ^b^ ± 0.04
**Testosterone (ng/ml)**	1.81 ^b^ ± 0.13	4.22 ^a^ ± 0.19

Values (Means ± SE) with different superscripts (a and b) in the same row are significantly different (*P* < 0.05) between rutting and non-rutting camels. TAC- Total antioxidant capacity MDA- Malondialdehyde

The correlations between concentration of TAC, MDA, and serum T, were analyzed in rutting and non-rutting camels (Table 1). In both rutting and non-rutting season the levels of T were found to be positively correlated with levels of TAC (*r* = 0.66, *p* < 0.01; *r* = 0.60, *p* < 0.01 respectively). However, levels of TAC were negatively correlated with levels of MDA during the non-rutting season (*r* = -0.59, *p* < 0.01).

**Table 2:** Correlation between serum testosterone, TAC, and MDA of testes in rutting and non-rutting camel

	Rutting		Non rutting	
	TAC	MDA	TAC	MDA
**MDA**	-0.44		-0.59**	
**T**	0.66**	0.38	0.60**	-0.01

**Mean values showed higher significance levels of correlation (*P* < 0.01)

TAC: total antioxidant capacity; MDA: malondialdehyde, T: testosterone

The testicular measurements of camel during rutting and non-rutting seasons are presented as mean ± SE in Table 2. In both the rutting and non-rutting seasons, the measurements of the left testis (length and width) were significantly (*p* < 0.05) larger than that of the right testis, while no significant differences between the left and right testis were found in testicular thickness.

**Table 3:** Testicular measurements (Mean ± SE) of male dromedary camels during rutting and non-rutting season

	Non-rutting season (Mean ± SE)			Rutting season (Mean ± SE)		
	Left	Right	*P* value	Left	Right	*P* value
**Testicular length (cm)**	7.24 ± 0.003	6.84 ± 0.01	0.0001	8.40 ± 0.10	7.31 ± 0.01	0.01
**Testicular width (cm)**	2.91 ± 0.01	2.34 ± 0.03	0.0013	4.81 ± 0.03	4.24 ± 0.02	0.0018
**Testicular thickness (cm)**	2.48 ± 0.02	2.45 ± 0.02	0.8325	2.87 ± 0.04	2.85 ± 0.03	0.36

The comparisons in testicular size (length, width, and thickness) throughout rutting and non-rutting seasons are given in Table 3. During the rutting season, testicular size (length, breadth, and thickness) was substantially greater than during the non-rutting season.

**Table 4:** Testicular measurements from male dromedary camels during rutting and non-rutting season

	Non-rutting season (Mean ± SE)	Rutting season (Mean ± SE)	*P* value
**Testicular length (cm)**	7.15 ± 0.09	7.90 ± 0.05	0.0149
**Testicular width (cm)**	2.34 ± 0.03	4.24 ± 0.02	0.0001
**Testicular thickness (cm)**	2.47 ± 0.02	2.86 ± 0.01	0.0008

### Histological and morphometrical analysis of camel testis

**Fig. 1:**
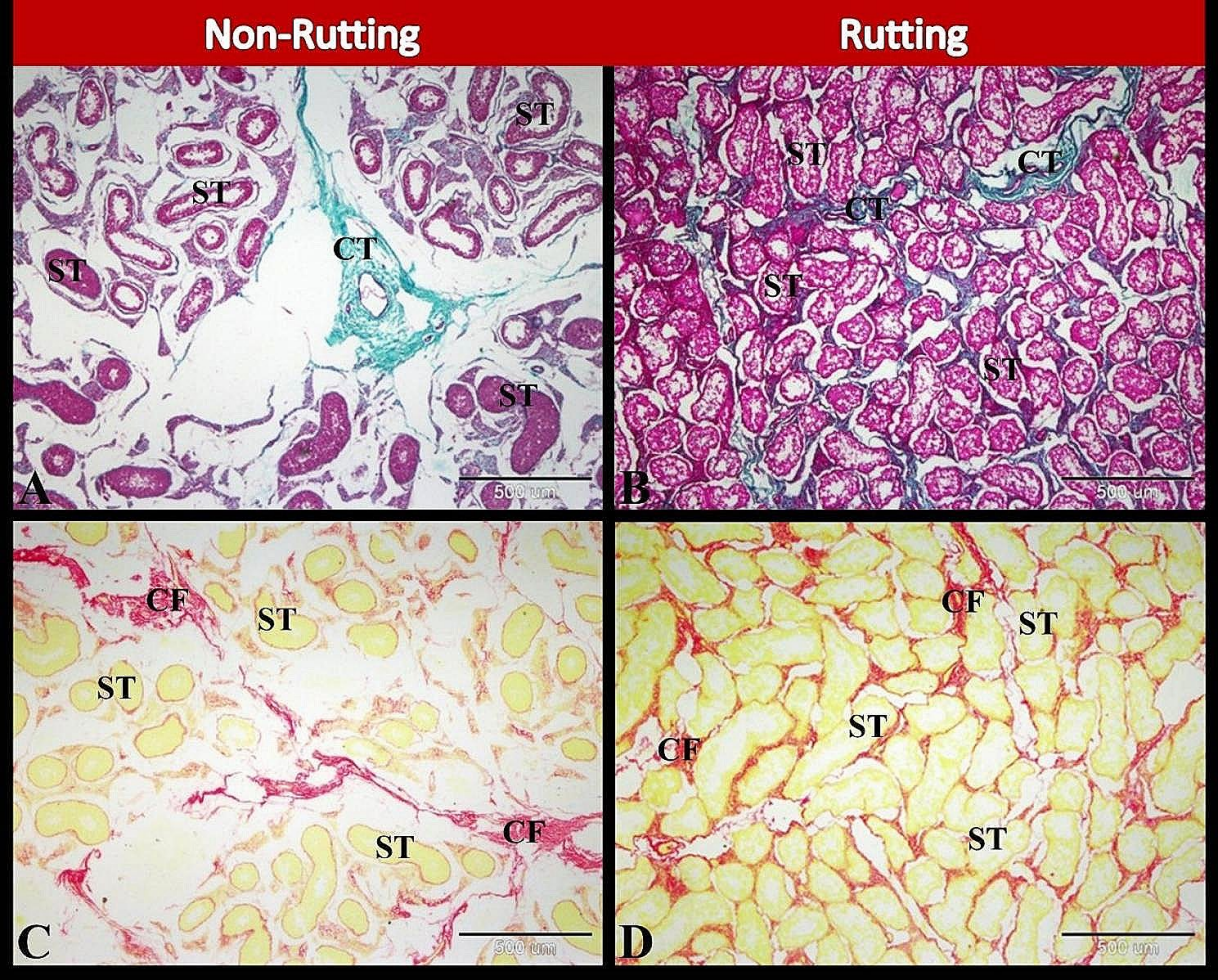
Photomicrographs of paraffin sections in camel testis during non-rutting (**A** & **C**) and rutting (**B** & **D**) seasons. A: Showing few seminiferous tubules (ST) surrounded by abundant interstitial and trabecular connective tissues (CT). B: Showing numerous seminiferous tubules (ST) surrounded by few interstitial and trabecular connective tissues (CT). C: Showing few seminiferous tubules (ST) surrounded by abundant collagen fibers (CF). D: Showing numerous seminiferous tubules (ST) surrounded by few collagen fibers (CF). Original magnification; A-D: X40, scale bar = 500 μm, A & B: Crossmon’s trichrome technique, C & D: Picro-Sirius red technique

**Fig. 2:**
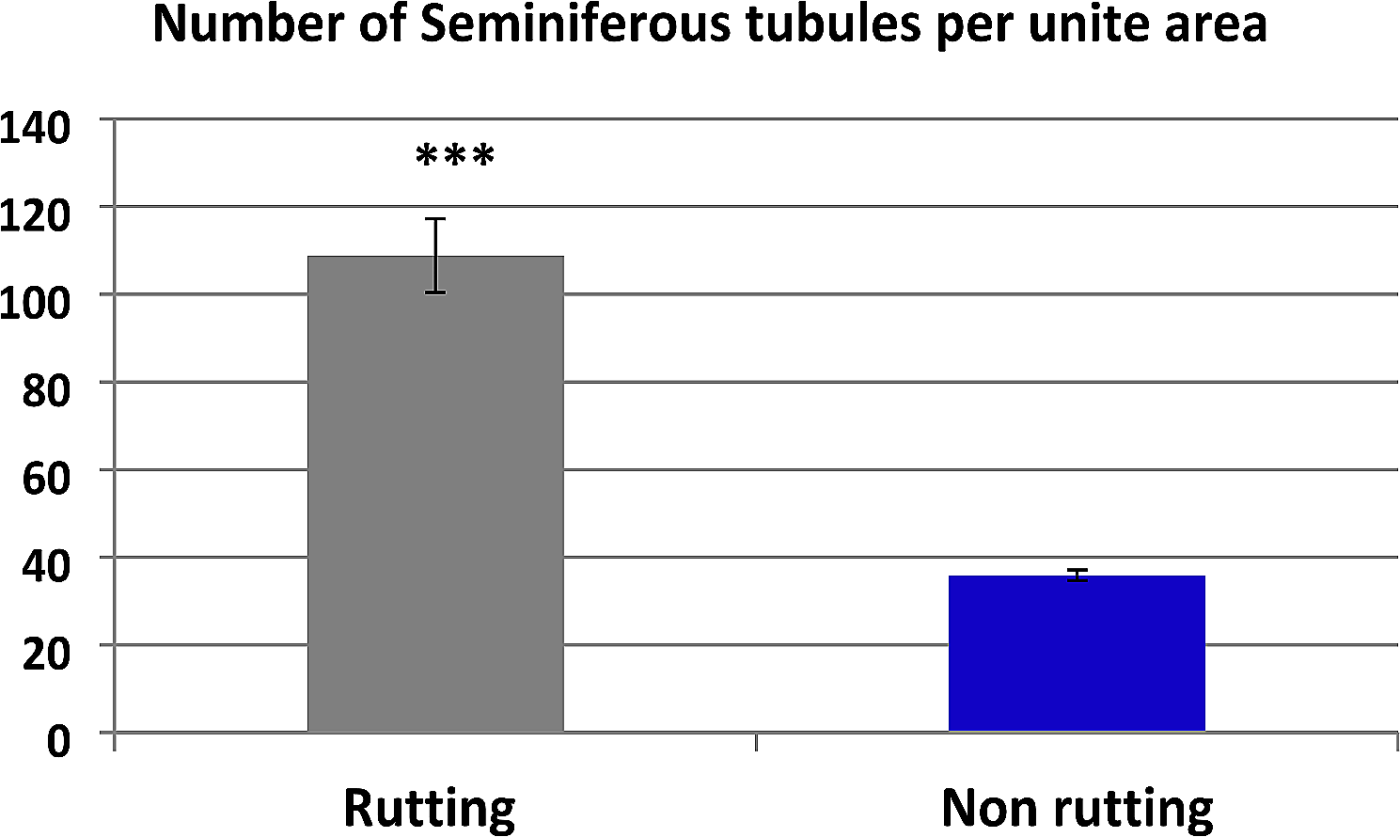
Showing the mean number of seminiferous tubules per unite area during rutting and non-rutting seasons

Histological and morphometrical evaluations of the camel testes during the non-rutting seasons revealed that the parenchyma of the testis was composed of a few small-sized seminiferous tubules surrounded by abundant interstitial and trabecular connective tissues formed of mature collagen fibers. However, the parenchyma of the testis during the rutting seasons was composed of numerous large-sized seminiferous tubules surrounded by a few interstitial and trabecular connective tissues formed of collagen fibers (Fig. 1A-D). It was found that the numbers and the size of seminiferous tubules significantly increased during the rutting season compared to non-rutting season (Table 5; Fig. 2).

**Table 5:** Mean diameter of seminiferous tubules and mean number of seminiferous tubules per unit area during rutting and non-rutting seasons

Season	Rutting (mean ± SE)	Non rutting (mean ± SE)	*P* value
Seminiferous tubules diameter (µm)	195.1^a^ ± 14.92	152.9^b^ ± 6.28	0.0048
Number of seminiferous tubules per unite area	108.8^a^ ± 8.4	35.9^b^ ± 1.2	0.0004

Values (Means ± SE) with different superscripts (a and b) in the same raw are significantly different (*p* < 0.05) between rutting and non-rutting camels

**Fig. 3:**
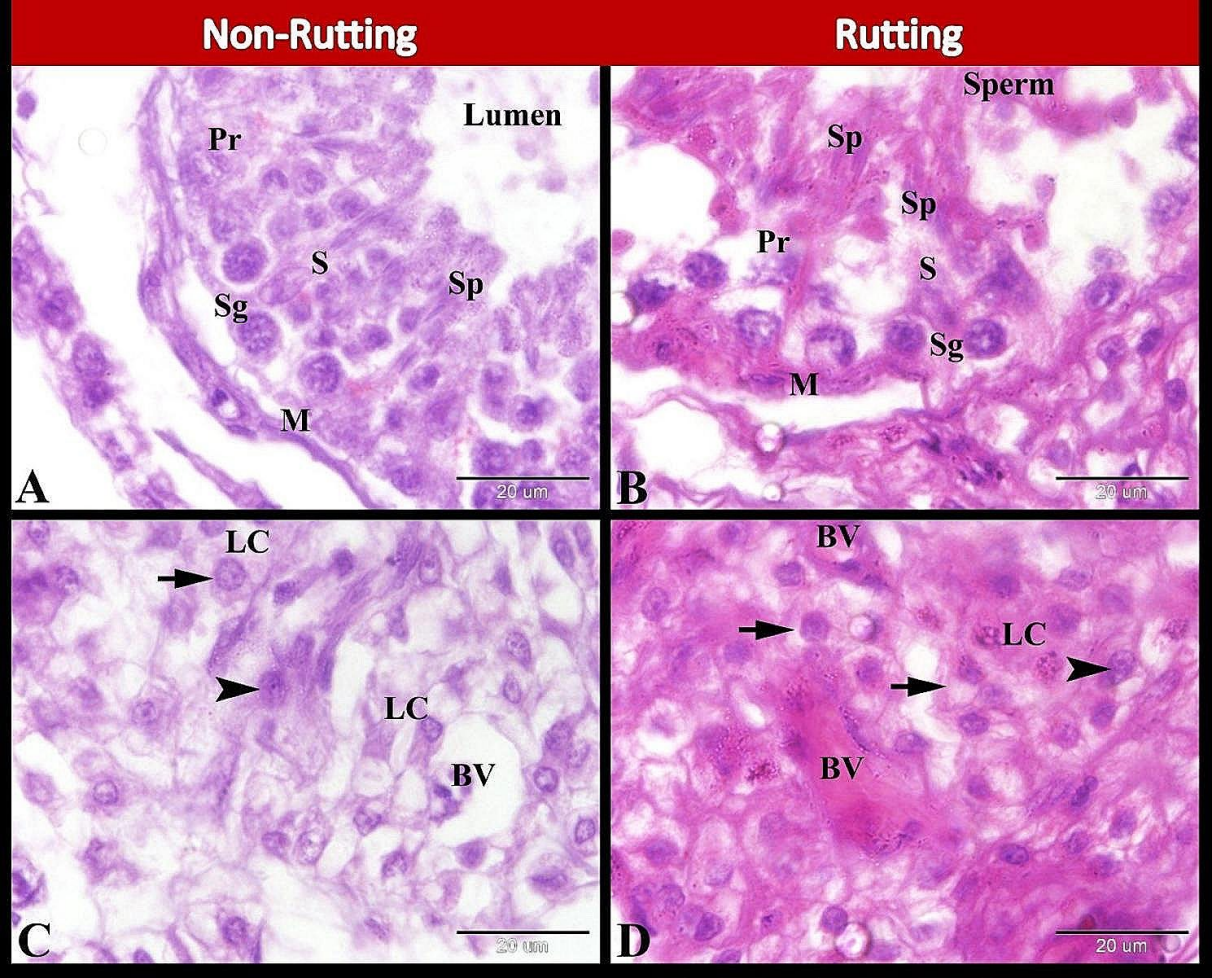
Photomicrographs of paraffin sections in camel testis during non-rutting (**A** & **C**) and rutting (**B** & **D**) seasons. A: Showing seminiferous tubule had a wide lumen and lined by stratified germinal epithelium and Sertoli cells (S). This germinal epithelium was formed of spermatogonia (Sg), primary spermatocyte (Pr), few spermatids (Sp) attached to the cytoplasmic processes at the apex of Sertoli cells (S). Note the myoid cells (M) concentrically arranged around seminiferous tubule. B: Showing seminiferous tubule had a narrow lumen filled with sperm and lined by stratified germinal epithelium and Sertoli cells (S). This germinal epithelium was formed of spermatogonia (Sg), primary spermatocyte (Pr), and many spermatids (Sp) attached to the cytoplasmic processes at the apex of Sertoli cells (S). Note the myoid cells (M) concentrically arranged around the seminiferous tubule. C: Showing Leydig cells (LC) which were polyhedral lightly stained vacuolated (arrow) epithelioid cells with a single centrally or eccentrically located rounded to ovoid nucleus (arrowheads) with prominent nucleoli. These cells are separated by a few blood vessels (BV). D: Showing Leydig cells (LC) which were polyhedral foamy eosinophilic (arrow) epithelioid cells with a single centrally or eccentrically located rounded to ovoid nucleus (arrowheads) with prominent nucleoli. These cells are separated by many blood vessels (BV). Original magnification; A-D: X1000, Scale bar A-D = 20 μm, Hx & E

Histological investigation of the camel testis during the non-rutting seasons revealed that the seminiferous tubules had a wide lumen and were lined by stratified germinal epithelium and Sertoli cells. This germinal epithelium was formed of spermatogenic cells in different stages of spermatogenesis. These spermatogenic cells were spermatogonia, primary spermatocytes, and a few spermatids. Spermatids were found attached to the cytoplasmic processes at the apex of Sertoli cells. Myoid cells were demonstrated concentrically arranged around seminiferous tubules (Fig. 3A). Whereas the seminiferous tubules during the rutting season had a narrow lumen filled with sperm and lined by stratified germinal epithelium and Sertoli cells. The seminiferous epithelium contained different spermatogenic cells; spermatogonia, primary spermatocyte, and many spermatids. Myoid cells were concentrically arranged around seminiferous tubules (Fig. 3B). Interestingly, we observed that during the non-rutting season, the interstitial cells of Leydig were polyhedral lightly stained vacuolated epithelioid cells with a single centrally or eccentrically located rounded to ovoid nucleus with prominent nucleoli. These cells were arranged in cords or clumps and separated by few blood vessels (Fig. 3C). While during the rutting season, the Leydig cells were polyhedral foamy eosinophilic epithelioid cells with a single centrally or eccentrically located rounded to ovoid nucleus with prominent nucleoli. These cells were separated by many blood vessels (Fig. 3D).

**Fig. 4:**
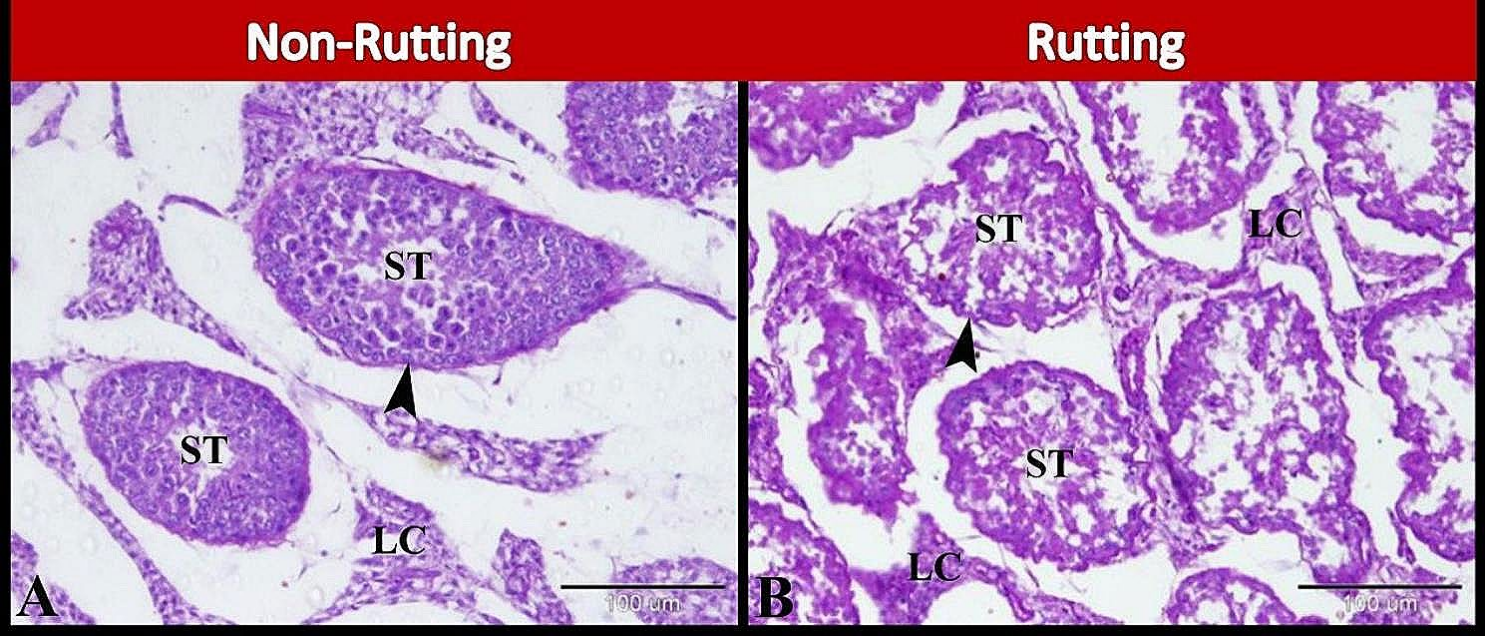
Photomicrographs of paraffin sections in camel testis during non-rutting (**A**) and rutting (**B**) seasons. A: Showing seminiferous tubules (ST) had nearly regular PAS-positive seminiferous basement membrane (arrowhead) B: Showing seminiferous tubules (ST) had irregular PAS-positive seminiferous basement membrane (arrowhead). Note Leydig cells (LC) between seminiferous tubules. Original magnification; A-B: X200, Scale bar A & B = 100 μm, PAS & Hx

Our results showed that during the non-rutting season, the seminiferous tubules had nearly regular PAS-positive seminiferous basement membrane (Fig. 4A). While during the rutting season, the seminiferous tubules had irregular PAS-positive seminiferous basement membrane (Fig. 4B).

**Fig. 5:**
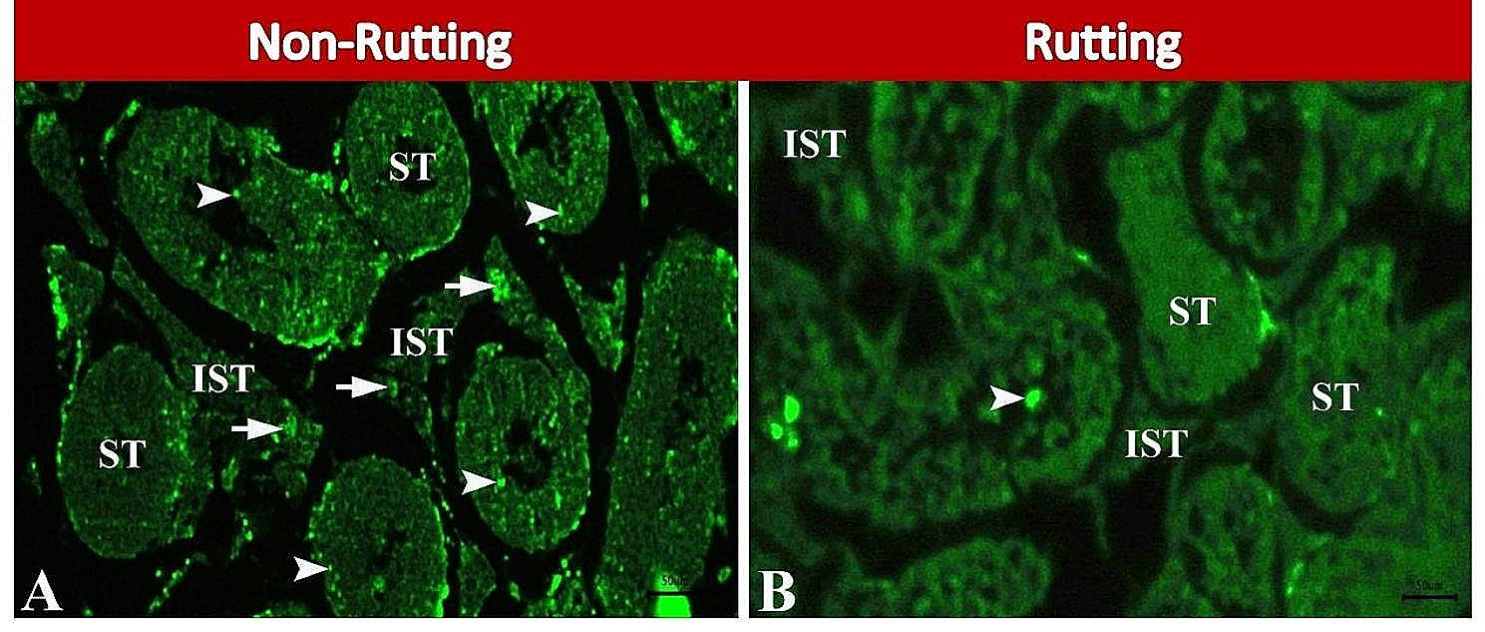
Photomicrographs of TUNEL assay of paraffin sections in camel testis during non-rutting (**A**) and rutting (**B**) seasons. A: Showing seminiferous tubules (ST) with many apoptotic spermatogenic cells (arrowheads) and interstitial tissues (IST) with many apoptotic Leydig cells (arrow). B: Showing seminiferous tubules (ST) with few apoptotic spermatogenic cells (arrowheads) and interstitial tissues (IST) with normal Leydig cells

Herein, apoptosis during non-rutting and rutting seasons was detected by TUNEL assay in paraffin sections of camel testis. We observed that there were many apoptotic spermatogenic cells and Leydig cells during non-rutting season (Fig. 5A). Few apoptotic spermatogenic cells and Leydig cells were demonstrated during rutting season (Fig. 5B).

## Discussion

The impact of season on androgen synthesis in the dromedary is quantitative and qualitative [3]. The prominent endocrine change in camel bull during the rutting season is an increase in the secretion of androgen and the spermatogenesis process [1]. Our finding in the current study noted that the highest level of serum testosterone occurred during the rutting period and is consistent with the findings of previous studies in camel bull confirming that the highest level of the testosterone hormone occurs during the rutting period [5, 18, 19].

The highest level of serum testosterone during the rutting season may be attributed to increased LH secretion from the pituitary gland or enhanced Leydig cell’s sensitivity to LH [18]. During the reproductive season, male dromedaries show greater sensitivity to GnRH stimulation with more significant LH pituitary release. Moreover, the rutting season affects LH plasma concentrations and increases its pulsatility [1].

The testosterone hormone is responsible for spermatogenesis, sperm maturation in the epididymis, and sexual behavior activity [19], and its low levels could impair normal spermatogenesis and lead to an increase in the percentage of spermatozoa with morphological abnormalities [20]. The beneficial effects of testosterone on male animals were described by [21] who reported an increase in serum testosterone levels in males positively affects their sexual activities. In addition, caput and cauda epididymal proteins were influenced by testosterone and some of these proteins are responsible for the improvement of spermatozoa maturation and storage [20].

The observed increase in the level of testosterone may be explained by the inhibiting influence of cortisol on testosterone production due to cortisol resulting in decreased LH receptors and testosterone production [2, 22]. The importance of an optimal level of testosterone in spermatogenesis is underscored by the fact that these hormones potently regulate vital sperm functions, capacitation, and acrosome reactions, and thus are responsible for the normal development of sperm cells [18, 23]. Reactive oxygen species (ROS) are generated during testosterone biosynthesis and the excessive production of ROS negatively affects male fertility [24]. The exposure of sperm to ROS causes DNA damage, membrane peroxidation, and decreased sperm motility. The presence of polyunsaturated fatty acids in the plasma membrane and low concentrations of cytoplasmic antioxidant enzymes make the sperm sensitive to oxidative stress [25].

An increase in ROS and reduction of antioxidants may lead to an increase in oxidative stress (OS) [26], which negatively affects semen quality, and induces detrimental effects on spermatozoa, which can cause lipid peroxidation in the sperm plasma membrane [27, 28]. Increased OS promotes sperm DNA damage and reduces sperm quality and fertility [28–30]. Malondialdehyde (MDA) is an oxidative stress-related marker and biomarker for membrane lipid peroxidation of omega-3 and omega-6 fatty acids [10]. Alternatively, the antioxidant system may confer sperm protection through increased antioxidants and reduce MDA, therefore constituting a defense mechanism against OS reactions [10, 31].

The determination of seminal TAC is a good indicator of the protection of sperm against damage caused by oxidative stress as well as a tool for the prognosis of fertility in buffalo bulls [32] and boars [33]. Measurement of MDA is commonly used as an indicator of sperm cell membrane damage by lipid peroxidation and analysis of fertility [32, 34]. However, the relationship between TAC and MDA levels in testis with camel fertility has not yet been reported. The highest TAC and lowest MDA levels were observed in the testes of the rutting group. This means that in the rutting season, sperm was protected from oxidative stress which is reflected in semen quality parameters including progressive vitality and abnormality.

The present study showed that during the rutting season, camel testes are larger (longer, wider, and thicker) than they are during the non-rutting season. These results are consistent with those of [35], who investigated the seasonal influence on camel testes’ size. Larger testicles are associated with an increase in the number, size, and activity of Leydig cells as well as higher development of interstitial tissues, which in turn lead to increased testicle size [8, 36]. The testicular measurements of the left testicle (length and width) in rutting and non-rutting seasons were significantly larger than those of the right testicles, similar results were found in earlier studies [37].

Concerning the numbers and the size of seminiferous tubules of camel bulls testes during the rutting and non-rutting season. The number and size of seminiferous tubules increased in the rutting season in the present study and similar findings have been reported earlier [38]. Moreover [39], in his study on donkey testicular sections during different seasons declared that during the breeding season, the tubular lumen of the seminiferous tubules was filled with many spermatozoa with a thicker germinal layer composed mainly from different forms of spermatogenic cells. Also, he observed a decreased size in donkey seminiferous tubules in the spring and summer seasons. The male dromedary camel’s testis during the breeding season consisted of many active seminiferous tubules of different sizes and shapes, with different maturation stages of spermatogenic cells observed as compared to non-breeding season [40].

There were many apoptotic spermatogenic and Leydig cells during non-rutting season as compared with rutting season in the present study which coincides with the finding of many degenerative changes with lowered numbers of mature cells in the summer season and this trend continued till the early and mid-autumn seasons [41].

## Conclusion

This study revealed that the rutting normal breeding season (NBS, rutting group) was associated with higher levels of total antioxidant capacity (TAC), T, and spermatogenic activity while the collagen content, concentrations of MDA (the oxidative stress factor), and apoptosis (an outcome of oxidative stress) were lower than those in the low breeding season (LBS, non-rutting group). In addition, the testicular size and seminiferous tubule diameter and numbers were higher during the NBS.

Interestingly, in both the rutting and non-rutting seasons the levels of T were positively correlated with the levels of TAC. However, the TAC was negatively correlated with the MDA content during the non-rutting season.

## Data Availability

The datasets used and/or analysed during the current study are available from the corresponding author on reasonable request.
